# Density-Induced Variations of Local Dimension Estimates for Absolutely Continuous Random Variables

**DOI:** 10.1007/s10955-025-03416-x

**Published:** 2025-02-15

**Authors:** Paul Platzer, Bertrand Chapron

**Affiliations:** Laboratoire d’Océanographie Physique et Spatiale (LOPS), Ifremer, 1625 route de Sainte-Anne, 29280 Plouzané, Bretagne France

**Keywords:** Local dimension, Information dimension, Attractor dimension, Random variable, Distribution, Curse of dimensionality, Weather regimes

## Abstract

For any multi-fractal dynamical system, a precise estimate of the local dimension is essential to infer variations in its number of degrees of freedom. Following extreme value theory, a local dimension may be estimated from the distributions of pairwise distances within the dataset. For absolutely continuous random variables and in the absence of zeros and singularities, the theoretical value of this local dimension is constant and equals the phase-space dimension. However, due to uneven sampling across the dataset, practical estimations of the local dimension may diverge from this theoretical value, depending on both the phase-space dimension and the position at which the dimension is estimated. To explore such variations of the estimated local dimension of absolutely continuous random variables, approximate analytical expressions are derived and further assessed in numerical experiments. These variations are expressed as a function of 1. the random variables’ probability density function, 2. the threshold used to compute the local dimension, and 3. the phase-space dimension. Largest deviations are anticipated when the probability density function has a low absolute value, and a high absolute value of its Laplacian. Numerical simulations of random variables of dimension 1 to 30 allow to assess the validity of the approximate analytical expressions. These effects may become important for systems of moderately-high dimension and in case of limited-size datasets. We suggest to take into account this source of local variation of dimension estimates in future studies of empirical data. Implications for weather regimes are discussed.

## Introduction

Local dimension estimation tools allow to study multifractal measures with local density exhibiting multiple scaling exponent. A first approach to study such measures is global, looking at these scaling exponents over the measure’s whole attractor, through what is called the spectrum of generalized dimensions [1–3]. The other approach is local, examining variations of estimated dimensions at different points of the attractor. Such an approach is widely used to study dynamical properties of atmospheric circulation [4–12], building on mathematical developments linking dynamical systems theory and extreme value theory [13]. These local dimensions allow to assess the probability distributions of distances for “analogs” [14], often used in atmospheric science for several applications (e.g. [15–18]). Local and global approaches can be reconciled, as [19] showed that the spectrum of generalized dimensions can be deduced from the ensemble of local dimensions estimates.

These dimension-estimation tools are designed for multifractal measures, and should in principle give trivial results when applied to random variables with smooth probability density functions. However, in practice, dimension estimates can be biased. [20] showed that in high dimension, the curse of dimensionality induces dimension estimates inferior to what is expected from the multi-fractal formalism of dynamical systems (i.e., that the local dimension should equal the phase-space dimension). It has also been noted that the dimension estimates are anomalously high in areas of low density [6], such as the borders of the wings of the three-variable convective Lorenz system [21].

In this work, we explore these seemingly intrinsic variations with position in phase-space of the estimates of local dimension for random variables possessing an absolutely continuous probability density function (hereafter referred to as “absolutely continuous random variables”). There is no physical reason to use multifractal analysis tools to study absolutely continuous random variables, which is why the present work must be motivated. First, the least to be expected from empirical numerical estimation tools is to recover trivial results anticipated for simple random variables. Second, the simple case of absolutely continuous random variables allows to derive analytical expressions for the possible biases of local dimension estimates when applied to such variables. Third, observations of physical systems such as the atmosphere (and its circulation) are affected by noise, so that empirical estimates of local dimension are computed on *blurred* multifractal dynamical systems that may share some properties with simple absolutely continuous random variables. For instance, [22] demonstrated the simple result that the local dimension of multifractal dynamical systems subject to additive noise equals the phase-space dimension (*i.e.* the dimension of the noisy process). Finally, our conjecture is that the results presented here for absolutely continuous random variables might be partially replicated for the more relevant case of simulated or observed multifractal systems.

In the present paper, we use Taylor expansions with the hyper-sphere-radius used to compute local dimensions, to derive analytical approximate expressions for the estimates of local dimension, leading to a typical formula that can be used to compute the latter from empirical data. These expressions are then compared to true empirical estimates of local dimension from numerically generated data corresponding to 1. a one-dimensional double-well stochastic system, 2. a two-dimensional Gaussian Mixture Model, and 3. a standard multivariate Gaussian of arbitrary dimension.

Section 2 recalls the basic definitions and provides analytical derivations for the approximate deviation of local dimension estimates from the phase-space dimension. Section 3 provides particular cases of the analytical expressions, and describes numerical experiments used to validate these expressions. Finally, Sect. 4 gives concluding remarks and discusses implications for studies of weather regimes based on dynamical indicators.

## Theoretical Background

### Definitions

Let us consider a dynamical system with invariant measure $\mu$. For any point *x* in the support of $\mu$ , the local, *r*-resolution dimension at point *x* follows:

1 $$\begin{aligned} d(x,r) := \frac{\log \mu (B_{x,r})}{\log r} \, , \end{aligned}$$

where $B_{x,r}$ is the ball of radius *r* centered on *x*, and $r>0$. The limit for small *r* of this local dimension, when it exists, is denoted *d*(*x*). If *d*(*x*) exists for all *x* and if $\mu$ is ergodic, then *d*(*x*) is constant $\mu$-almost everywhere and the system is said to be exact-dimensional [23]. In this case the typical value of *d*(*x*) is noted $D_1$ and is called the first-order Renyi dimension or information dimension. It is also equal to the $\mu$-average of the local dimensions :

2 $$\begin{aligned} D_1 := \lim _{r\rightarrow 0}\frac{\int \log \left( \mu (B_{x,r}) \right) d\mu (x)}{\log r} \, . \end{aligned}$$

[19] showed that the local dimensions *d*(*x*, *r*) follow a large deviation principle around their $\mu$-average value $D_1$ as $r\rightarrow 0$. This gives information on the probability density of *d*(*x*, *r*) when $|d(x,r)-D_1|$ exceeds a given threshold.

### Numerical Estimation

We assume that we are provided with a long time-series of $\left\{ x_i \right\} _{1\le i\le N}$ from the dynamical system defining $\mu$, where *N* is a large integer.

Computing *d*(*x*, *r*) at fixed *x* through Eq. (1) with a Birkhoff sum to estimate $\mu (B_{x,r})$ gives a slow convergence to $D_1$ for small values of *r*. Instead, methods relying on $\mu ( B_{x,r})$ for several small values of *r* give more satisfying results. Let $K\in \mathbb {N}$, and let $r_1< \ldots < r_K$ the ordered distances to the *K* nearest neighbors of *x* in the dataset $\left\{ x_i \right\} _{1\le i\le N}$. Then the following expression is an estimator of $d(x,r_K)$ (see [18]):

3 $$\begin{aligned} \hat{d}(x,r_K) := \left\{ \sum _{k=2}^K \frac{k}{K} \log \left( \frac{r_k}{r_{k-1}} \right) \right\} ^{-1} \, . \end{aligned}$$

For practical purpose, the number of neighbors *K* must be chosen adequately. On the one hand, *K*/*N* should be small enough to ensure small bias of the estimator $\hat{d}(x,r_K)$, *i.e.* the latter must approach $D_1$ on an average taken over possible datasets $\left\{ x_i \right\} _{1\le i\le N}$, each dataset being sampled as one trajectory of the same dynamical system (for instance, with different initial conditions). On the other hand, *K*/*N* should be large enough to ensure small variance of the estimator $\hat{d}(x,r_K)$, *i.e.* the latter should have a small variance taken over possible datasets sampled as mentioned. In the right-hand side of Eq. (3), the sum is an approximation of:

4 $$\begin{aligned} \sum _{k=2}^K \frac{k}{K} \log \left( \frac{r_k}{r_{k-1}} \right) \approx \int _0^{r_K}\frac{\mu (B_{x,r})}{\mu (B_{x,r_K})} \frac{\textrm{d}r}{r} \, \end{aligned}$$

by using the approximation $\log \left( \frac{r_k}{r_{k-1}} \right) \approx \frac{r_k-r_{k-1}}{r_k}$, which is valid only when $\frac{r_k-r_{k-1}}{r_k}$ is small. Note that from [18], we have the scaling $r_k \sim k^{1/D_1}$, so that the previous approximations holds in particular for medium-to-large values of the dimension, and for medium-to-large values of *k*. For instance, if $D_1=1$ and $k=1$, we have the scaling $\frac{r_k-r_{k-1}}{r_k}\sim 1$ and so this approximation barely holds. On the contrary, if $D_1=3$ and $k=4$, then $\frac{r_k-r_{k-1}}{r_k}\sim 0.07$. For practical applications such as large-scale atmospheric flows [6], the dimension is usually larger than 6 and one uses more than 100 neighbors for the estimation of local dimensions, so that the proposed approximation is justified.

Note also that, if we assume that there are constant values $d,\,\mu _0>0$ such that, for $r<r_K$, $\mu (B_{x,r}) = \mu _0 r^d$, then the integral in the right-hand side of Eq. (4) equals exactly $d^{-1}$. Equation (4) allows to directly reassess $\hat{d}(x,r_K)$ as a function of *r*:

5 $$\begin{aligned} \hat{d}(x,r) \approx \left\{ \int _0^{r}\frac{\mu (B_{x,r'})}{\mu (B_{x,r})} \frac{\textrm{d}r'}{r'} \right\} ^{-1} \, . \end{aligned}$$

In the following, we will focus on the behavior of $\hat{d}(x,r)$ using this expression.

### Expansion for Absolutely Continuous Random Variables

#### Fixed Radius

In this section, the attractor measure $\mu$ describes the probability of an absolutely continuous random variable, i.e. the following formula is true for any *n*-dimensional phase-space volume *V*:

6 $$\begin{aligned} \mu (x\in V) = \int _V p(x) \textrm{d}^n x \, \end{aligned}$$

where *p*(*x*) is the probability density function of the random variable, and a smooth function of *x*. We also assume that *p*(*x*) has no zeros, and no singularity (*i.e.* for all *x*, $0<p(x)<+\infty$). Note that this key hypothesis is a strong one, as several physically relevant systems are described by densities with singularities (see examples in [24, 25]), and relaxing this hypothesis allows to build systems that exhibit a continuous spectrum of generalized dimensions although they are absolutely continuous random variables (as in *e.g.* [26]). Based on this hypothesis, the quantity $\mu (B_{x,r})$ admits a Taylor expansion for small *r*:

7 $$\begin{aligned} \mu (B_{x,r})&= \int _{B_{0,r}} p(x+u) \textrm{d}^n u \end{aligned}$$

8 $$\begin{aligned}&= \int _{B_{0,r}} \left\{ p(x) + \nabla p (x) \cdot u + \frac{1}{2} u \cdot \left[ H(p)(x) \, u \right] + \mathcal {O}(u^3) \right\} \textrm{d}^n u \, \end{aligned}$$

where $\nabla p(x)$ denote the *n*-dimensional gradient of *p* at *x*, and *H*(*p*)(*x*) denotes the $n\times n$ - dimensional Hessian matrix of *p* at *x*, the matrix of second-order derivatives, and centered dot “$\cdot$” denotes scalar product.

The first term in the integral is constant and gives $p(x)\alpha _n r^n$ where $\alpha _n>0$ is the volume of a radius-1, *n*-dimensional ball. Through symmetry in the ball $B_{0,r}$ the integral $\int _{B_{0,r}} u \textrm{d}^n u$ of the odd function $u\mapsto u$ vanishes and so does the second term in the integral. Finally the third term can be re-written as a sum of odd and even functions.

9 $$\begin{aligned} \int u \cdot \left[ H(p)(x) \, u \right] \textrm{d}^n u =\sum _{i\ne j} \partial _i \partial _j p \int u_i u_j \textrm{d}^n u + \sum _i \partial _i^2 p \int u_i^2 \textrm{d}^n u \, . \end{aligned}$$

In this expression, terms that depend on cross-derivatives along different directions vanish, and the sum of non-vanishing terms amounts to $\frac{1}{2n} \Delta p(x) \beta _n r^{n+2}$ where $\Delta p(x) = \sum _i \partial _i^2 p(x)$ is the Laplacian of *p* at *x* (i.e. the trace of the Hessian matrix) and $\beta _n$ is the integral $\int _{B_{0,1}} u^2 \textrm{d}^n u$. Through vanishing integral of odd functions the fourth term of order $\mathcal {O}(u^3)$ (non-written here) also vanishes, so that one can write:

10 $$\begin{aligned} \mu (B_{x,r}) = p(x) \alpha _n r^n + \frac{\beta _n}{2n} \Delta p(x) r^{n+2} + \mathcal {O}(r^{n+4}) \; . \end{aligned}$$

Coming back to $\hat{d}(x,r)$, one can also estimate the following integral as:

11 $$\begin{aligned} \int _0^r\frac{\mu (B_{x,r'})}{r'}\textrm{d}r' = \left( \frac{1}{n}\right) p(x) \alpha _n r^n + \left( \frac{1}{n+2}\right) \frac{\beta _n}{2n} \Delta p(x) r^{n+2} + \mathcal {O}(r^{n+4}) \; , \end{aligned}$$

which gives, after manipulation, and using the fact that $(n+2)\beta _n = n\alpha _n$:

12 $$\begin{aligned} \hat{d}(x,r) = n \left\{ 1 + \frac{\Delta p(x)}{p(x)} \left( \frac{r}{n+2}\right) ^2 \right\} + \mathcal {O}(r^4) \, . \end{aligned}$$

This final expression shows that, for absolutely continuous attractor measures $\mu$, the first order deviations of $\hat{d}(x,r)$ from the exact, integer phase-space dimension *n* is of order $r^2$. The Laplacian of *p*(*x*) is positive (resp. negative) in case of local minima (resp. maxima) of probability. This means that in highly sampled areas, the estimated dimension decreases, while around poorly sampled areas, the estimated dimension increases. However, this effect is weighted by a factor $p(x)^{-1}$, so that the increase of estimated dimension near poorly sampled area is enhanced, while the decrease of estimated dimension near highly sampled areas is mitigated. In particular, the positions of extrema of $\hat{d}$ differ from those of *p* in general.

In one dimension, Eq. (12) reads:

13 $$\begin{aligned} \hat{d}(x,r) = 1 + \frac{ \partial _x^2 p(x) }{9 p(x)} r^2 + \mathcal {O}(r^4) \, . \end{aligned}$$

where $\partial _x^2p(x)$ is the second-order derivative of *p* at *x*. In two dimensions (*x*, *y*), we have:

14 $$\begin{aligned} \hat{d}((x,y),r) = 2 + \frac{ (\partial _x^2+\partial _y^2) p(x,y) }{8\, p(x,y)} r^2 + \mathcal {O}(r^4) \, . \end{aligned}$$

More generally, one can check that when taking the $\mu$-average of $\hat{d}(x,r)$, we have:

15 $$\begin{aligned} \int _\Omega \frac{\Delta p(x)}{p(x)} \; \textrm{d}\mu&= \int _\Omega \Delta p(x) \textrm{d}^n x \end{aligned}$$

16 $$\begin{aligned}&= \int _{\delta \Omega } \nabla p(x) \cdot \textrm{d}^{n-1}x \, , \end{aligned}$$

where the last integral is the flux of the gradient of *p* at the border of the whole domain $\Omega$, which is zero. This gives:

17 $$\begin{aligned} \int \hat{d}(x,r) \textrm{d}\mu (x) = n + \mathcal {O}(r^4) \; , \end{aligned}$$

which is a low-order particular case of the general statement that the $\mu$-average of local dimensions is the order-1 Renyi dimension (here *n*).

Note that these computations could be extended to the case where *p*(*x*) describes random variables taking values on discrete sets such as $\mathbb {N}^n$. In this case, *p* is not a function but a distribution [27], more precisely *p* is a sum of Dirac-delta functions, so that integrals involving *p* can be expressed as finite sums. If *p* is a discrete approximation of a smooth function of $\mathbb {R}^n$, our results could be extended straightforwardly. Otherwise, more efforts are needed to give a simple yet general expression of $\hat{d}(x,r)$ for the case of variables taking values in discrete sets.

#### Fixed Quantile

In practice, when trying to compute local dimensions, one rarely fixes the radius *r*, but rather the quantile *q* which is the proportion of data used to compute the local dimensions. Indeed, fixing the radius can become complicated when data are poorly sampled, as this would imply to rely on very few points for computing *d*.

The quantile *q* can be related to the radius and probability density function by noting that $q = \mu \left( B_{x,r} \right)$ by definition, and recalling Eq. (10), which gives at first order:

18 $$\begin{aligned} r = \left( \frac{q}{p(x)\alpha _n} \right) ^\frac{1}{n} \left( 1 + \mathcal {O}\left( \frac{r^2}{n}\right) \right) \; . \end{aligned}$$

Inserting this in Eq. (12) gives:

19 $$\begin{aligned} \hat{d}(x,q) \approx n \left\{ 1 + \frac{\Delta p(x)}{p(x)^{1+2/n}} \frac{(\Gamma (\frac{n}{2}+1)q)^{2/n}}{\sqrt{\pi }(n+2)^2} \right\} \, , \end{aligned}$$

where $\Gamma$ is the Gamma function that enters into the expression of the volume of a radius-1, *n*-ball: $\alpha _n = \pi ^{n/2}/\Gamma (n/2+1)$. In the case of large *n*, one can recover Eq. (17) as the second-term of the right-hand side of Eq. (19) is still approximately proportional to $\Delta p(x)/p(x)$.

Equation (19) exhibits a more complex relationship with *n* compared to Eq. (12), emphasizing how *r* depends on *n* when *q* is held constant. However, an additional dependence on dimension is hidden in the ratio $\Delta p(x)/p(x)$, since probability density functions are also significantly influenced by dimension. For instance, the probability density function of a standard normal vector evaluated at 0 decreases with dimension *n* as $(2\pi )^{-n/2}$. Particular cases are outlined below to better understand these expressions.

## Particular Cases and Numerical Experiments

### Double-Well Potential

First consider a one-dimensional example of a stochastic system emanating from the following stochastic differential equation (SDE, see e.g. [28]):

20 $$\begin{aligned} \textrm{d}x = -\partial _x V(x) \textrm{d}t + \sigma \textrm{d}W \, , \end{aligned}$$

where *x*(*t*) is real-valued, *t* is time, $\sigma >0$ and *W* is a Wiener-process of variance d*t*, with the following potential:

21 $$\begin{aligned} V(x) = (1-x^2)^2 \; , \end{aligned}$$

which is the famous symmetric double-well. In particular, this potential has the following drift and second-order derivative:

22 $$\begin{aligned} -\partial _x V = 4(1-x^2)x \; , \end{aligned}$$

23 $$\begin{aligned} \partial _x^2 V = 4(3x^2-1) \; . \end{aligned}$$

This potential has two stable equilibrium points at $x=\pm 1$ and one unstable equilibrium point at $x=0$. We have $\partial _x^2 V(\pm 1)=8$, and $\partial _x^2 V(0)=-4$.

The Fokker–Planck equation associated with the above SDE is:

24 $$\begin{aligned} \partial _t p(x,t) = \partial _x\left[ p(x,t)\partial _xV(x) \right] + \partial _x^2\left[ \frac{\sigma ^2}{2} p(x,t) \right] \; , \end{aligned}$$

which has the static solution :

25 $$\begin{aligned} p_s(x) = \frac{\exp \left( -\frac{2V(x)}{\sigma ^2} \right) }{\int _{-\infty }^{+\infty }\exp \left( -\frac{2V(u)}{\sigma ^2} \right) \textrm{d}u} \; . \end{aligned}$$

**Fig. 1:**
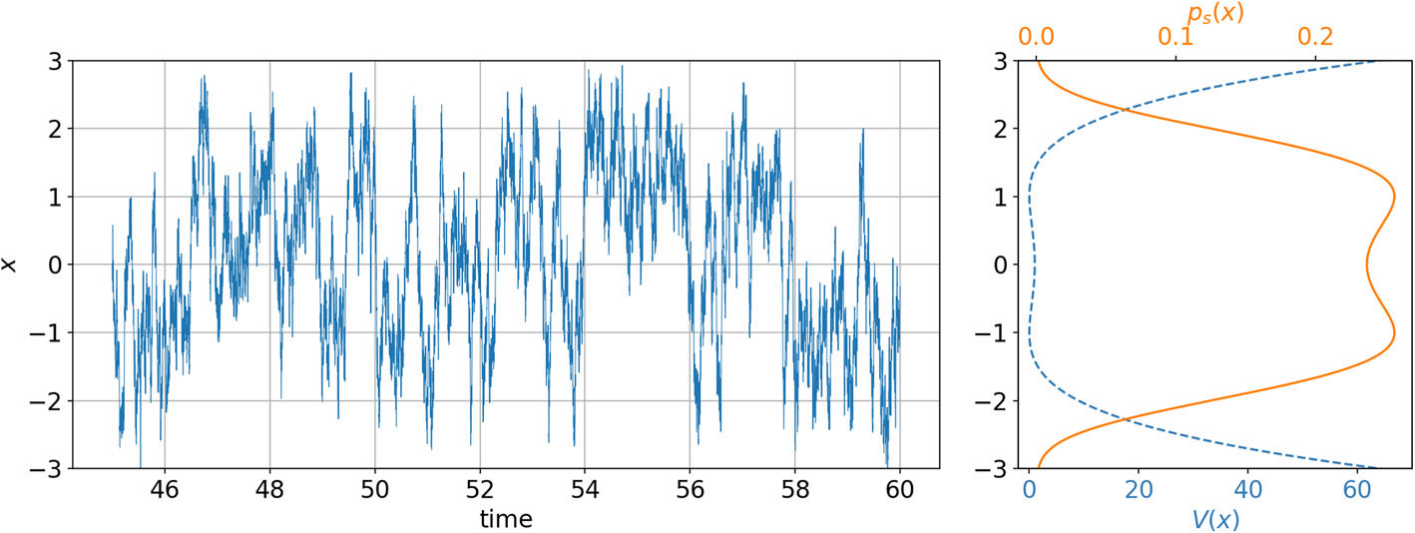
Left: example of trajectory following Eq. (20) with potential (21), and noise amplitude $\sigma =5$. Right: corresponding potential (dashed blue line) and static probability density (full orange line) (Color figure online)

One simulation of this stochastic system with the Euler–Maruyama method and a time step of $10^{-3}$ is shown in the left panel of Fig. 1. The right panel shows the corresponding potential and static probability density function. We can see the typical behavior of this system, jumping randomly from one well to another.

According to the derivations of Sect. 2.3, dimension estimates are expected to deviate from the true dimension $n=1$, with lower dimensions around the wells of the potential, and higher dimensions not only at the centered unstable fixed point but also to the right and left of the wells. More precisely, combining $p_s(x)$ from Eq. (25), expressed from Eq. (21), with Eq. (13) gives:

26 $$\begin{aligned} \hat{d}(x,r) = 1 + \frac{8}{9\sigma ^2}\left[ 1-3x^2+\frac{8}{\sigma ^2}(1-x^2)^2 x^2\right] r^2 + \mathcal {O}(r^4) \; . \end{aligned}$$

**Fig. 2:**
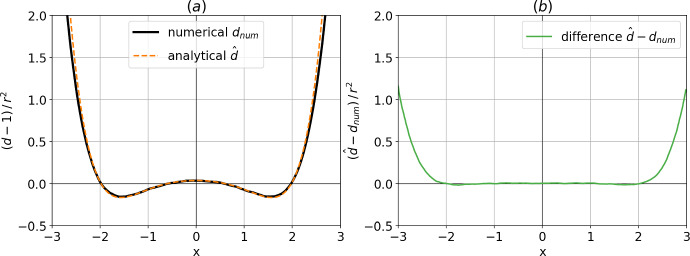
**a** Numerical estimate of local dimension $d_{num}$ from a long simulation of the double-well stochastic system (black, full) and analytical approximation $\hat{d}$ for this estimate (dashed, orange) as a function of the phase-space position *x*. For both curves, we substract the true dimension 1 and normalize by $r^2$ the square of the radius used to compute local dimensions. **b** Difference between the two curves (Color figure online)

A numerical simulation of Eq. (20) is performed with time step $10^{-3}$, running for $5\times 10^5$ non-dimensional time. This numerical simulation will serve as a “catalog” from which the distances $r_k$ are computed. The empirical local dimension is then estimated on a regular grid. The interval $-3<x<3$ is spanned, using Eq. (3) at fixed radius $r=0.3$ by choosing *K*(*x*) at each position *x* so that $r_K<r<r_{K+1}$, and the $\lbrace r_k \rbrace _k$ are the distances between *x* and the elements of the catalog. These empirical estimates of $\hat{d}(x,r)$ are then compared with the approximate analytical expression Eq. (26), and shown in Fig. 2. The approximation appears to be valid for the whole interval considered, although it overestimates the true numerical estimates for $|x|\gtrsim 2.5$. Note that the approximation still captures an interesting feature, also present in the empirical estimates of dimension from the simulated catalog: the position of the minimum of dimension differs from that of the maximum of probability.

This last point is of importance for the recurring, quasi-stationary patterns of atmospheric circulation called “weather regimes” (WRs, see [29–31]). Weather regimes are well known to weather forecasters because of their frequency and impact on local weather [32, 33]. One example of winter-time WR in the North-Atlantic region is the positive phase of the North-Atlantic oscillation, which can be roughly described as the co-occurrence of a low-pressure cyclonic system over Iceland and a high-pressure anticyclonic system over the Azores. This regime is observed several times every winter and brings stormy weather from the North-Atlantic ocean to western Europe [34, 35]. Although the undeniable chaotic nature of atmospheric circulation [36] suggests that WRs should be interpreted as a particular feature of a complex dynamical system, one mostly uses statistical tools to study WRs, rather then dynamical systems metrics. In particular, the empirical study of WRs relies on the fit of atmospheric circulation data^1^ to a Gaussian mixture model^2^ (GMM, see *e.g.* [41]), each average of the GMM allowing to define a different regime. Yet, recent studies [6, 8, 11, 42] have shown that dynamical systems metric such as the local dimension bear distinguished features associated to WRs, even when the latter are defined from a statistical GMM fit. This fact was argued to be a clue that the dynamical and statistical views of weather regimes could be reconciled. More specifically, it was shown that estimates of local dimension *decrease* when atmospheric circulation projection onto GMM means is *maximal* [8, 42], while estimates of local dimension *increase* near transitions between GMM-defined regimes [11]. This behavior could arguably be the feature of a dynamical system whose local dimension increases near transitions from one fixed point to another, as observed in the Lorenz system [21] (see the supplementary material of [6]). However, the present investigation shows that even purely stochastic systems with absolutely continuous measures could also bear this feature of change of estimated local dimension at the transition between metastable states. Since Fig. 2 reveals that the positions of the maxima of probability differ from those of the minima of dimension, it appears that the relationship between density and bias in estimated dimension is non-trivial, and should be examined further for applications to weather regimes. In the next subsection, we investigate on a simple example how this density-induced effect of local dimension estimates acts on two-dimension Gaussian mixture random variables. These experiments will provide hints of how to use the present work for the numerical study of multifractal properties of weather regimes.

**Fig. 3:**
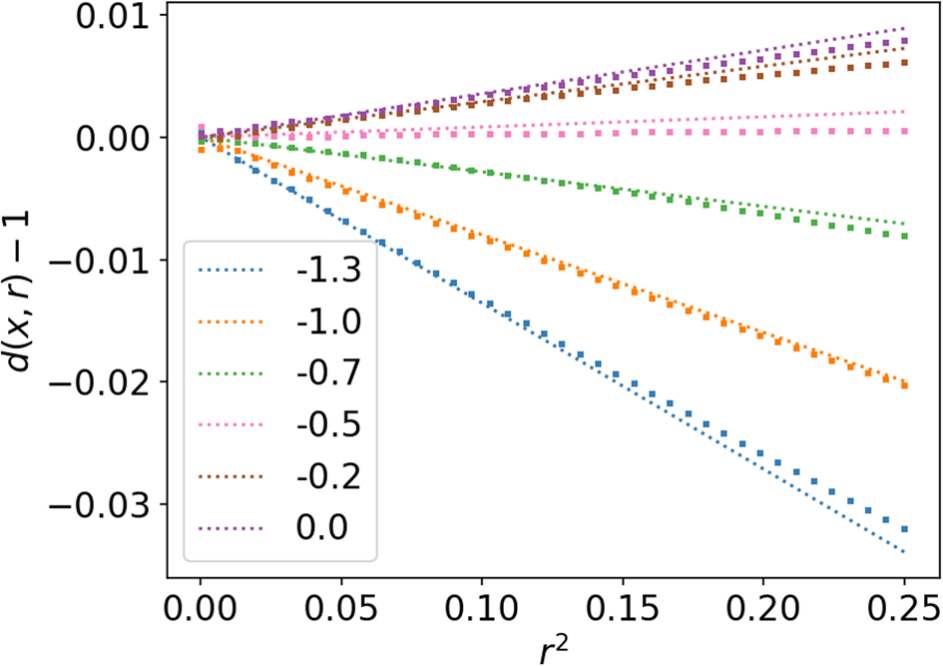
Local dimension estimate as a function or $r^2$ for several locations *x* in the double-well stochastic system, estimated from numerical simulations compared to an analytical approximation. Squares: approximation from Eq. (26). Dotted lines: empirical values from numerical simulation

This first simple one-dimensional example can finally be used to investigate one property of Eq. (26), which is the scaling $\hat{d}(x,r)-1 \sim r^2$. To do so, we take a closer look at a few points *x* between −1.3 and 0 for which the approximation seems to be valid according to Fig. 2. We take regularly sampled values of $r^2$ between 0.0004 and 0.25, for which the local dimension is estimated with Eq. (3), and *K* defined as previously through $r_K<r<r_{K+1}$. These estimates are compared with the analytical expression of Eq. (26) in Fig. 3. The agreement between the analytical approximation and the empirically estimated values is satisfying, validating both the scaling of $\hat{d}(x,r)-1$ with $r^2$ and the values of the slopes given by the analytical expression $\frac{ \partial _x^2 p(x) }{9 p(x)}$ in dimension 1.

### Gaussian Mixture Model

The previous example showed that the position of minima of dimension differs from that of the maxima of probability density. To test this assertion, a Gaussian Mixture Model is considered (GMM, see e.g. [41]), of which k-means [40] are a particular case. Such a model allows to define a random variable as stemming from several components (of the “mixture”), each component being defined by a Gaussian distribution with its own characteristics (mean and covariance matrix).

As mentioned in the previous section, these statistical models are also typically used to assign atmospheric circulation data to weather regimes. For instance, in [31], four weather regimes are defined through the fit of a GMM to atmospheric circulation data, smoothed in time and projected in a two-dimensional subspace. As a reminiscence of this configuration, we take here interest in a two-dimensional random variable defined by a GMM with four components. This random variable *X* has the following distribution $p_X$:

27 $$\begin{aligned} p_X = \sum _{i=1}^4\phi _i \mathcal {N}(\varvec{m_i,\Sigma _i}) \; , \end{aligned}$$

where $\mathcal {N}(\varvec{m,\Sigma })$ stands for the probability density function of a Gaussian distribution with mean $\varvec{m}$ and covariance matrix $\mathbf {\Sigma }$, and each $\phi _i$ corresponds to the probability of a given component to be selected. We choose to use diagonal covariance matrices for simplicity. The values set for the means $\varvec{m_i}$, covariances $\varvec{\Sigma _i}$ and probabilities $\phi _i$ are listed in Table 1.

**Table 1:** Parameters used for the two-dimensional Gaussian Mixture Model

Parameter $\backslash$Component	Upper-right	Lower-right	Lower-left	Upper-left
$\varvec{m_i}$	[1.5 , 1.5]	[1 , −1]	[−1 , −0.9]	[−1 , 0.9]
$\varvec{\Sigma _i}$	1.3	0.9	1.2	0.9
$\phi _i$	0.25	0.25	0.25	0.25

**Fig. 4:**
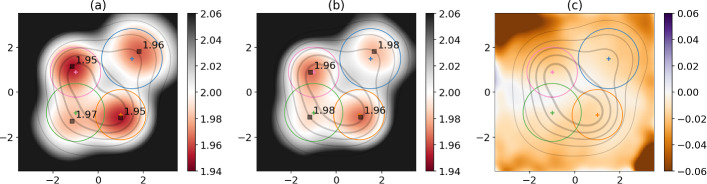
In all three panels are displayed both the GMM density (contours) and the Gaussian distributions’ averages (crosses) and radius at which the probability of the Gaussian is twice smaller then its maximum probability (circles). **a** Approximate analytical expression for the dimension estimates from Eq. (14) and true GMM density, setting radius $r=0.5$. Squares indicate the position of the theoretical minima of dimension estimates, and the corresponding minimum values of dimension are written next to the squares. **b** Same as (**a**) but for the empirical dimension estimates from numerically sampled GMM and with Eq. (3) at fixed radius $r=0.5$. To produce panel (**b**), a Gaussian filter was applied to the two-dimensional image of local dimension estimates with a bandwidth of 0.25 in terms of the positions *x* of the GMM. **c** Difference between panels (**a**) and (**b**) : analytical minus (smoothed) empirical dimension estimate

Covariance matrices are proportional to the identity matrix and therefore only one coefficient is given. The components are given names related to their position in phase-space, as shown in the plots of Fig. 4

Although feasible, there is no interest in giving the analytical expression for the approximate expression of $\hat{d}(x,r)$ from Eq. (14) using the expression for the probability density function of this random variable. However, we can visually check the agreement between this analytical expression and the true estimates of dimension from numerical experiments. To do so, we draw $10^7$ samples of the GMM, and for each two-dimensional position *x* on a regular grid of $200\times 200$ points ranging from $-3.5$ to $+3.5$ in both dimensions, we compute the empirical dimension at radius $r=0.5$ using this randomly sampled data and Eq. (3). Since the resulting numerically estimated local dimension is too noisy for an automatic detection of local minima, we further apply a two-dimensional Gaussian filter with a bandwidth of 0.25 to the numerically estimated local dimensions (using the function gaussian_filter from the python package scipy.ndfilters). The result of this procedure is shown in Fig. 4b, and compared to our approximate analytical expression in Fig. 4a, while the third panel Fig. 4c shows the difference between the two. There is a reasonably good agreement between our approximation and the empirical estimates in terms of the position of the minima of estimated dimension, as well as the general behavior, including rising dimension in areas of low density, far from the GMM components. Our approximation also predicts that the position of the minima of dimension slightly differ from the GMM means, with an offset towards higher values of |*x*|, similarly to the one-dimensional double-well example. Our approximation is lower than the numerical estimates of local dimension over the whole area considered, and the largest discrepancies are found far from the center of the distribution, as in the previous one-dimensional example. However, the agreement is sufficient to draw conclusions on the global shape and order of magnitude of the numerically estimated dimensions (Fig. 4b) using our approximation (Fig. 4a).

This example shows that our approximation captures anomalous variations in estimated dimension for random systems stemming from a GMM. In particular, troughs of dimension are witnessed near the regime means, so that the observations of [8, 11, 42] may indeed be associated to such effects of density variation rather than changes in the fractal properties of the attractor of the underlying atmospheric dynamical system. However, the amplitude of the variations in dimension estimate observed in this example are small, and more investigations are needed to understand how these effects depend on phase-space dimension. This is the subject of the following particular case.

### Multivariate Gaussian

A multivariate Gaussian system is now considered, to more particularly explore the effect of dimensionality on our claims. For a standard multivariate Gaussian, the probability density function is given by:

28 $$\begin{aligned} p(x) = \frac{\exp \big (-\frac{|x|^2}{2}\big )}{(2\pi )^{\frac{n}{2}}} \; , \end{aligned}$$

where *x* is a *n*-dimensional vector. The Laplacian of *p*(*x*) reads:

29 $$\begin{aligned} \Delta p(x) = \left( |x|^2 - n \right) \frac{\exp \big (-\frac{|x|^2}{2}\big )}{(2\pi )^{\frac{n}{2}}} \; . \end{aligned}$$

Substituting this into Eq. (19) gives:

30 $$\begin{aligned} \hat{d}(x,q) \approx n \left\{ 1 + 2 \sqrt{\pi } \left( |x|^2 - n \right) \exp \left( \frac{|x|^2}{n}\right) \frac{(\Gamma (\frac{n}{2}+1)q)^{2/n}}{(n+2)^2} \right\} \, . \end{aligned}$$

Again, the witnessed behavior is similar to those depicted in the previous experiments, with a decreased estimated dimension towards the area of high probability density (here $x=0$), and an increased estimated dimension with respect to the theoretical value *n* in areas of low density (here for large values of |*x*|).

**Fig. 5:**
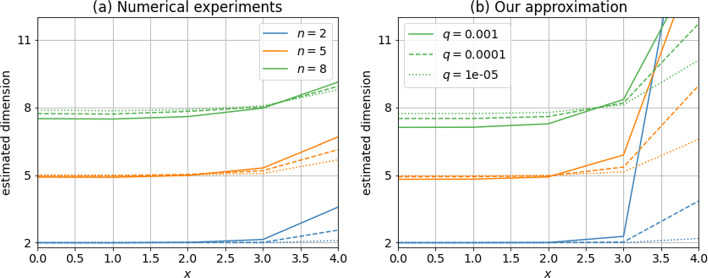
Estimated local dimension for the multivariate normal distribution, at positions $x=(0,0,\ldots ,0)$, $x=(1,0,\ldots ,0)$, up to $x=(4,0,\ldots ,0)$, for three values of *q*, the proportion of data used to compute the local dimensions, and three values of *n*, the exact dimension. **a** Values obtained from numerical experiments, averaged over 100 realisations for each triplet (*x*, *q*, *n*). **b** Approximation from Eq. (30)

These formulas are only approximations of the true behavior of local dimension estimates for data generated from the standard multivariate Gaussian. To test their validity using numerical experiments, we first consider five different positions $x=(0,\ldots ,0)$, $x=(1,0,\ldots ,0)$, $x=(2,0,\ldots ,0)$, $x=(3,0,\ldots ,0)$, $x=(4,0,\ldots ,0)$; as well as three values of the proportion of data used to compute the local dimensions $q=10^{-3}$, $q=10^{-4}$, $q=10^{-5}$; and finally three values for the exact dimension $n=2$, $n=5$, $n=8$. To each triplet of values (*x*, *q*, *n*) can be associated an approximate value of $\hat{d}(x,q)$ from Eq. (30), which is reported in Fig. 5b. To compare this to real dimension estimates from numerical experiments, we generate 100 independent datasets for each pair (*q*, *n*), each dataset containing $10^3/q$ samples of the standard multivariate Gaussian of exact dimension *n*. Then, with each dataset we compute the local dimension estimate using Eq. (3) at all five positions *x* listed above. For each triplet (*x*, *q*, *n*), we therefore obtain 100 values for $\hat{d}(x,q)$. Taking the average over all 100 realisations, we obtain the results shown in Fig. 5a.

Numerical experiments confirm the same tendency as the ones given by our approximate Eq. (30): slightly lower than *n* for $x=0$, and growing with |*x*|, eventually exceeding *n*. The deviation at $x=0$ from the exact value *n* is a growing function of *n* for the values considered here, both according to our approximation and to the numerical experiments. At fixed position $x=4$, the opposite behavior is observed: the deviation is stronger for small values of *n*. Note also that, as in the previous experiments, our approximation strongly departs from the numerical values in areas of very small density (here, large values of |*x*|). Our approximation also overestimates the deviation at $x=0$. However, these numerical experiments display the same behavior as one would expect from our approximation, suggesting that the latter adequately represents the effect of changing density on variations of numerical estimates of local dimension.

**Fig. 6:**
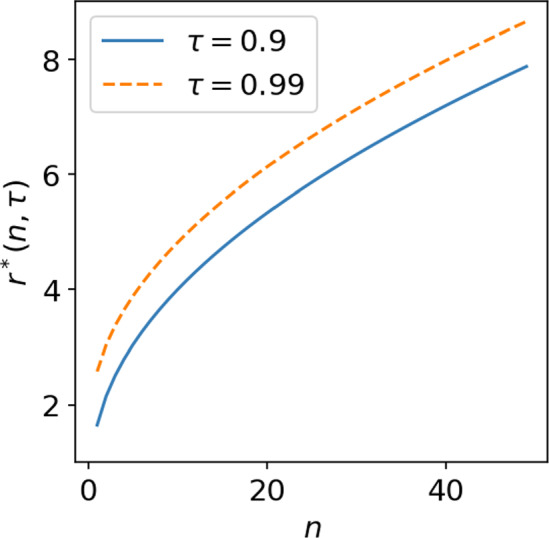
Plot of radius $r^*(n,\tau )$ versus phase-space dimension *n* defined through Eq. (31). This is the radius for which the probability of a standard multivariate Gaussian to lie in a 0-centered ball of radius $r^*$ equals $\tau$

A next question is then the following: what is the typical variation of local dimension estimates that is only due to density variations, and how does this typical variation depend on the exact dimension *n* ? To test this, we define the radius $r^*(n,\tau )$, where $0<\tau <1$ is the probability that *x* lies in a ball of radius $r^*$ centered on $x=0$. This radius $r^*$ is thus defined implicitly through the following equation:

31 $$\begin{aligned} \tau = (r^*)^{n-1} \exp \left( \frac{(r^*)^2}{2} \right) \; , \end{aligned}$$

where the right-hand side of this equation is obtained by integrating the probability density function of a standard Gaussian distribution from $r=0$ to $r=r^*$. We can find an approximate value for $r^*(n,\tau )$ by solving this equation numerically for each desired values of *n* and $\tau$: see Fig. 6 for the behavior of $r^*$ with *n*.

Then, our objective is to estimate the following quantity:

32 $$\begin{aligned} \frac{\Delta \hat{d}}{n} := \frac{1}{n}\left( \hat{d}(|x|=r^*(n,\tau ),q) - \hat{d}(|x|=0,q) \right) \; . \end{aligned}$$

**Fig. 7:**
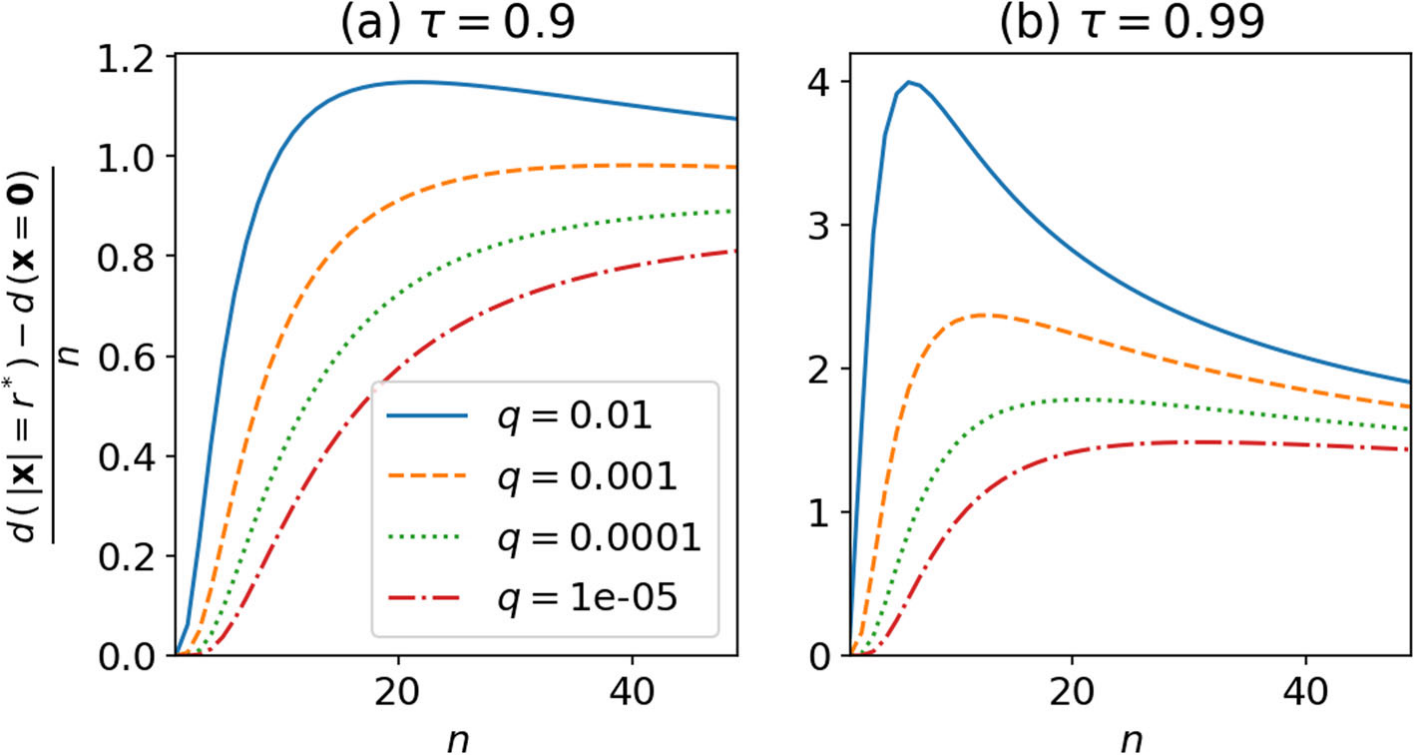
Typical variations of dimension estimates for the multivariate Gaussian, as predicted from our approximation Eq. (30), as a function of phase-space dimension *n*, and for various values of the ratio *q* of total data used to compute the local dimensions. **a** Probability $\tau =0.9$ of being in a centered ball of radius $r^*$. **b** Same with $\tau =0.99$

This last quantity is representative of the typical variations of fractal dimension estimate that would be observed roughly $1-\tau$ times on average. These variations are only due to changes in probability density, and they do not not represent variations in fractal properties. Since there is no analytical expression for $r^*$, we cannot give an explicit expression for $\frac{\Delta \hat{d}}{n}$ using our approximation (30), however we can plot it numerically. This is shown in Fig. 7. Our approximation (30) predicts that $n\mapsto \frac{\Delta \hat{d}}{n}$ is a growing function of *n* for $\tau =0.9$ and values of *q* below 0.001, while it reaches a maximum for moderate values of *n* if $\tau =0.9$ and $q=0.01$, or if $\tau =0.99$ and for all considered values of *q*. This maximum value of $n\mapsto \frac{\Delta \hat{d}}{n}$ depends on *q* and $\tau$, as well as the value of *n* for which the maximum is reached. Fig. 7 indicates very strong values for $\Delta \hat{d}$, up to 4 times the exact phase-space dimension *n*. On the one hand, noting the discrepancy between our approximation and numerical experiments from Fig. 5, we expect these numbers to greatly overestimate the true value of $\frac{\Delta \hat{d}}{n}$. On the other hand, the good agreement shown in Fig. 5 between experiments and analytical approximation in terms of behavior with *n* and *q* suggests that the same kind of qualitative agreement could be found for $\frac{\Delta \hat{d}}{n}$.

**Fig. 8:**
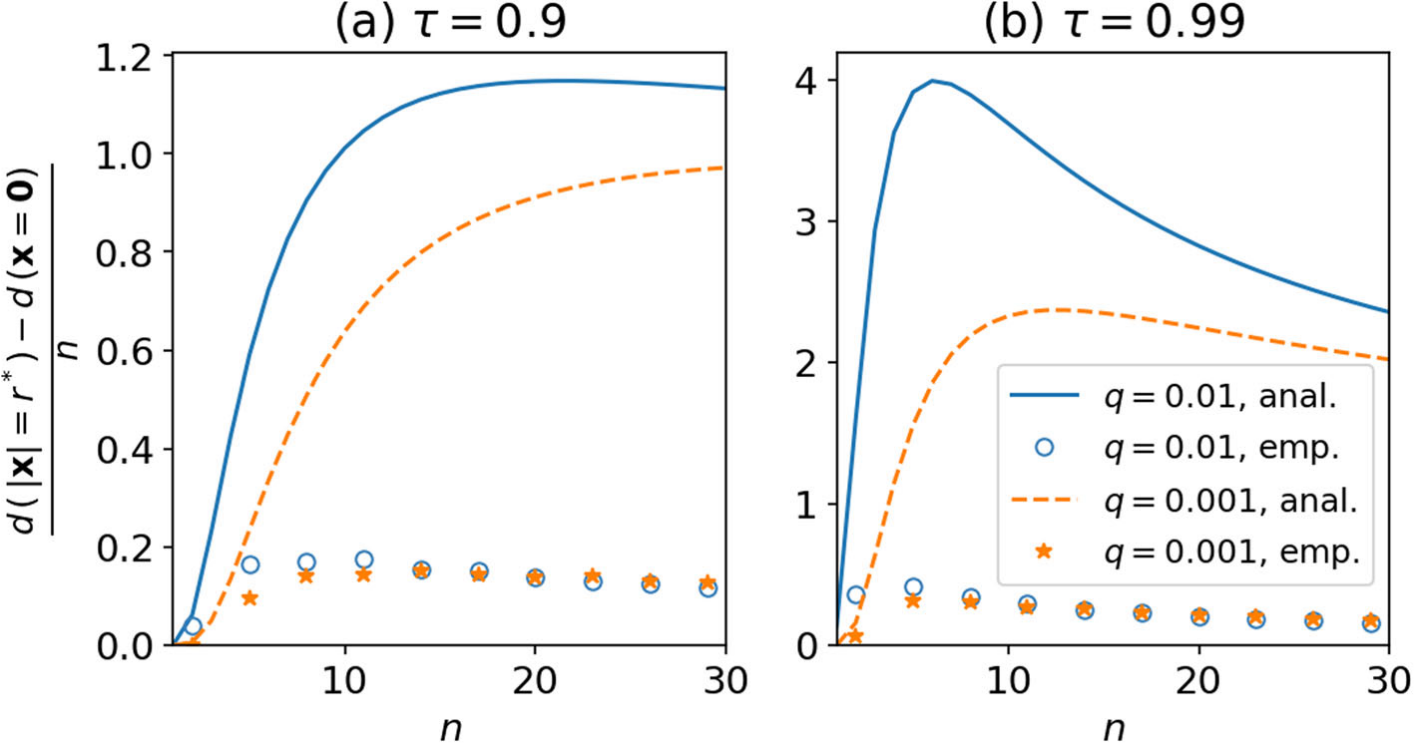
Estimated variations of local dimension $\frac{\Delta \hat{d}}{n}$ for the multivariate normal distribution, between position $x=(0,0,\ldots ,0)$, and $x=(r^*,0,\ldots ,0)$, where $r^*$ is such that the probability to be within distance $r^*$ from $x=0$ is $\tau =0.9$, for two values of *q*, the proportion of data used to compute the local dimensions, and 10 values of *n*, the exact dimension. Circles and stars: values obtained from numerical experiments, averaged over 100 realisations for each triplet $(\tau ,q,n)$. Full lines: approximation from Eq. (30), exactly as in Fig. 7

To test the validity of these approximations, numerical experiments are again performed. This time, we use two values for $q=10^{-3} \, , \, 10^{-4}$; and ten values for $n=2\, , \, 5 \, ,\, 8 \, ,\, \ldots \, , \, 29$; and finally two values for $\tau =0.9\, ,\, 0.99$. As in the previous experiment, for each pair (*q*, *n*) 100 independent datasets are generated, each containing $10^3/q$ samples of the standard multivariate Gaussian of exact dimension *n*. For each dataset, Eq. (3) is used to estimate the dimension at $x=(0,0,\ldots ,0)$ and at $x=(r^*,0,\ldots ,0)$. For each triplet $(\tau ,q,n)$, 100 values are therefore obtained for $\frac{\Delta \hat{d}}{n}$. Taking the average over all 100 realisations, we obtain the empty circles and full stars shown in Fig. 8, and compared against the semi-analytical curves of the previous figure.

The comparison with the approximation from (30) confirms that the latter strongly overestimates the amplitude of these variations, by approximately one order of magnitude. However, as in the previous experiment, the behavior is quite the same between our theoretical approximation and the numerical experiments. For $n=2$, $\frac{\Delta \hat{d}}{n}$ is negligible. The numerical estimates of $n \mapsto \frac{\Delta \hat{d}}{n}$ at fixed $(q,\tau )$ seem to reach a maximum between $n=5$ and $n=8$ for $q=10^{-2}$, while the maximum is reached for slightly higher values of *n* in the case $q=10^{-3}$. The empirical values of the maxima are $\sim 0.15$ for $\tau =0.9$ (one event out of 10) and between $\sim 0.3$ and $\sim 0.4$ for $\tau =0.99$ (one event out of 100) depending on the value of *q*. Although our approximation overestimates these effects, they are still non-negligible in practice. According to this experiment, for a system of dimension $n=14$, and using 1 millionth of the data to compute local fractal dimensions, the difference in estimated dimension between the most probable position ($x=0$) and a position which is visited one time out of 10 (respectively 100) is of the order of 15% (respectively 30%) of the true dimension, that is $\sim 2$ (respectively $\sim 4$).

To recall, a dimension of 14 is typical of continental-scale atmospheric circulation systems [6]. This experiment suggests that variations of local dimension estimates of the order of 2–4 might be due to changes in local density, and not to true changes in the fractal dimension. Note that such an amplitude of local variations of dimension is usually interpreted as variations in the local fractal nature of the attractor [6]. The results shown here suggest that these variations may be more difficult to interpret, possibly embedded with changes associated to uneven sampling of the phase-space caused by local changes in density.

## Conclusion and Perspectives

Approximate analytical expressions have been derived to anticipate the variations of local dimension estimate of random variables possessing an absolutely continuous measure (i.e., a continuous probability density function) without zeros or singularities. Such variables should not display variations of the local dimension according to the multi-fractal formalism of dynamical systems. These variations are therefore not related to the local fractal properties of the attractor. Rather, they are consequences of uneven sampling of the phase-space due to local changes in density of the underlying system. The derived approximate analytical expressions are compared to numerical experiments, proving relevant for a one-dimensional double-well stochastic system, a two-dimensional Gaussian Mixture Model, and finally standard multivariate Gaussian random variables. Although the given approximations overestimate these anomalous variations, good qualitative agreement is found between the behavior expected from our approximations and that observed in the numerical simulations.

The issue tackled in this work is related to that of [20], who showed that the attractor dimension, obtained by averaging local dimensions on the attractor estimated as in Eq. (3), differs from the true phase-space dimension for random variables with absolutely continuous measures. Here, we focused not on the average of the local dimension but on the variations of this local dimension. [20] showed that the deviation from the true phase-space dimension *n* of the averaged local dimension is strongest in high-dimension and with low values of *q*, the proportion of data used to compute the local dimension. Here, studying the *relative* variations in phase-space of local dimension for a multivariate Gaussian (see Eq. 30), we find similar results for the dependency on *q*. However, since we focus on the relative variations of dimension estimates, we expect the variations of local dimension to be strongest for moderate values of the phase-space dimension *n*, around $n\sim 11$ (see empirical values of Fig. 8). For atmospheric circulation data with typical local dimensions between 8 and 13 [6], our results suggest that such density-related effects could, *in principle*, be the prominent drivers of dimension estimate variability for these studies.

Furthermore, tests on simulated Gaussian Mixture Model data also suggest that the effect of lower dimension around regime peaks (as observed by [8] and [42]), and higher dimension around transitions between regimes (observed by [11]), is also obtained for purely random systems that should not, in principle, exhibit local variations of the local attractor dimension. However, note that this is only true if the regime peak happens close to the center of the regimes. On the contrary, [42] showed that the effect of lowered dimension around regime peaks is strongest for high value of the peak weather-regime index, *i.e.* far from the regime centers, where the density of data is low. According to our work, such a behavior is not expected for random variables with absolutely continuous measures, because the latter would witness an increase of dimension far from regime centers due to the lower data density. This last fact strengthens, on the contrary, the idea that the observed diminution of local dimension around peak weather regime index is dominated by effects of change in the multi-fractal nature of the attractor, rather then the density-based effects studied here.

It must be noted here that weather regimes offer a very specific view into atmospheric dynamics, as they are usually computed after two operations: 1. temporal smoothing (*e.g.* with a 10-days running average) 2. projection onto leading empirical orthogonal functions^3^. These two operations have a smoothing effect on atmospheric dynamics. This leads to a modification of the attractor by suppressing small-scale processes. It was shown that by looking at large time-scales one is able to recover the underlying attractor structure even when the system (or its simulation/observation) is subject to noise [44]. It was also shown that time-smoothing increases the estimated dimension of atmospheric dynamics [45]. These observations suggest that the smoothing operations undertaken before the weather regime analysis should be able to unveil the chaotic attractor of large-scale atmospheric dynamics. Therefore, the local dimension estimates associated with weather regimes should differ significantly from what is expected for a purely random system. We are currently working on a methodology to test this assertion, building “stochastic twins” of atmospheric dynamics that are purely random but share a density function which is close to that of observed atmospheric dynamics.

These elements suggest that more investigations are needed to establish the relevance of these results to real atmospheric circulation from realistic model simulations and observations. Taking these inquiries further would allow to assess the relative importance of two concurring views of weather regimes: the statistics-based description which views atmospheric circulation as a random system subject to fluctuations between different metastable states, and the dynamical systems-based description where local variations in the fractal properties of the attractor drive the dynamics of the system.

More broadly speaking, this study suggests that at least a part of the variability of estimated dimension fluctuations is due to changes in density, and not solely changes in fractal properties. Being able to discriminate the part of estimated dimension variability related to each of these two sources would allow one to interpret better the notion of dimensionality from such estimates. In particular, with the objective of building a low-order model, one would be interested in knowing if the largest values of estimated dimension are due to changes in fractal properties (in which case a large number of variables would be needed in a low-order model) or to changes in density (in which case one could rely on a number of variables lower than the largest estimated dimension). Again, further developments are needed in order to separate these two sources. For multi-scale dynamical systems, combining local dimension estimation tools with scale-decomposition algorithms [44] could be used to decipher the influence of density-changes and scaling-exponent-changes on the local dimension estimates computed on the whole system with all scales combined. Indeed, for such systems the ratio of the different sources of variation of local dimension estimate are likely to be very much scale-dependent, and one could use this property to isolate each source of variation of local dimension estimates.

## Data Availability

The code used to generate data and produce figures for this article is accessible upon request.

## Footnotes

1 Weather regimes are typically studied from “reanalysis” data such as the famous ERA5 [37], which provides reliable estimates of atmospheric circulation from the late 1970s-on thanks to global coverage from satellite observations [38] combined with other observation sources and with physics-based numerical weather prediction models [39].

2 One can either use Gaussian mixture models or a “k-means” algorithm [40], which makes no difference from a statistical standpoint.

3 Empirical orthogonal functions form a linear basis of the phase-space and are estimated based on a singular value decomposition of the symmetric positive time-covariance matrix of the data. It is used extensively in atmospheric sciences [43].
